# Engineering a Thermostable Reverse Transcriptase for RT-PCR Through Rational Design of *Pyrococcus furiosus* DNA Polymerase

**DOI:** 10.3390/biom15111507

**Published:** 2025-10-24

**Authors:** Aleksandra A. Kuznetsova, Irina A. Grishina, Elena S. Mikushina, Nikita A. Kuznetsov

**Affiliations:** 1 Institute of Chemical Biology and Fundamental Medicine, Siberian Branch of Russian Academy of Sciences, Novosibirsk 630090, Russiai.grishina@g.nsu.ru (I.A.G.);; 2 Department of Natural Sciences, Novosibirsk State University, Novosibirsk 630090, Russia

**Keywords:** reverse transcriptase, DNA polymerase, PCR, *Pyrococcus furiosus*, mutagenesis, rational design

## Abstract

Engineering of a bifunctional enzyme that combines DNA-dependent DNA polymerase and reverse transcriptase (RT) activities is a highly promising biotechnological goal, as it would enable one-enzyme RT-PCR. For this purpose, we selected the high-fidelity *Pyrococcus furiosus* (Pfu) DNA polymerase as engineering scaffold. The selection of amino acid residues for replacement was carried out based on a multi-sequence alignment of diverse DNA polymerases and literature data, which allowed us to target amino acids, which presumably are triggers of the RT activity appearance. Six mutant variants of the Pfu enzyme were created and their activity was analyzed. Through enzymatic screening, we identified the Pfu-M6 variant, which exhibits dual DNA-dependent and RNA-dependent DNA polymerase activity. This thermostable enzyme retains its inherent DNA polymerase function and has acquired the ability to catalyze reverse transcription under standard PCR conditions, which allows the created mutant form to be used for efficient amplification of DNA starting from an RNA template.

## 1. Introduction

DNA-dependent DNA polymerases are essential enzymes responsible for DNA replication, repair, and recombination in all living organisms. They are indispensable tools in molecular biology, genetic engineering, and molecular diagnostics, particularly in the polymerase chain reaction (PCR) [1,2,3]. The growing availability of engineered thermostable DNA-dependent DNA polymerases with enhanced properties such as processivity, synthesis rate, fidelity, and resistance to inhibitors has expanded their applications beyond basic research into pharmaceuticals, medicine, and diagnostics [4]. Consequently, the continuous search for novel enzymes with improved capabilities remains a priority. A key focus of this search is the development of RNA-dependent DNA polymerases, or reverse transcriptases (RTs), engineered from the robust scaffolds of DNA-dependent DNA polymerases. Since RTs’ discovery in retroviruses, these enzymes have been crucial for creating complementary DNA (cDNA) libraries from RNA templates that enable the sequencing of RNA genomes as well as perform gene cloning for subsequent expression of recombinant proteins.

Natural RTs are typically heat-labile and lack the proofreading 3′→5′ exonuclease activity that corrects mis-incorporated nucleotides. The absence of proofreading activity results in a high error rate of approximately 10^−4^ (1 error per 10,000 incorporated nucleotides). Given the widespread use of RT in generating cDNA for research and diagnostics, enhancing its efficiency and accuracy is a critical objective [5]. A promising solution is the engineering of a single enzyme that combines DNA-dependent and RNA-dependent DNA polymerase activities, which is highly desirable for one-enzyme RT-PCR. As the most accurate thermostable DNA polymerases belong to the B family [6], they have been used as scaffolds to create novel, high-fidelity RTs [5,7,8]. Through directed evolution [9,10] and site-directed mutagenesis [11,12], mutant B family DNA polymerases have been developed that not only function as efficient reverse transcriptases but can also utilize modified nucleic acids (XNAs) as templates [7]. The concept of “scaffold sampling” based on screening gain-of-function amino acid residues in homologous enzymes and their substitution in the target enzyme allowed us to obtain engineered DNA polymerases with non-DNA polymerase activity [13].

The high-fidelity DNA polymerase from *Pyrococcus furiosus* (Pfu) [14] was selected for the present study. Crystal structure of Pfu, solved at 2.6 Å resolution [15], reveals a canonical B family architecture. The 775-amino acid polypeptide is organized into five domains: an N-terminal domain (residues 1–130, 327–368), a 3′→5′ exonuclease domain (131–326), a palm domain (369–450, 501–588), a finger domain (451–500), and a thumb domain (589–775). In the absence of DNA, Pfu adopts an open conformation where the fingers and thumb domains are rotated outward by 33° and 24°, respectively. DNA binding induces a transition to a closed conformation, in which the thumb and fingers subdomains move toward the palm, resulting in tight substrate binding.

The proofreading 3′→5′ exonuclease domain excises mis-incorporated nucleotides by hydrolyzing their phosphodiester bonds. Incorporation of a non-complementary nucleotide causes local helix destabilization, facilitating the translocation of the DNA terminus into the exonuclease active site for cleavage. This process is only possible when the thumb subdomain is open. Coordination between the polymerase and exonuclease sites is presumably achieved through interaction of an exonuclease domain loop (residues 144–158) with a positively charged region of the thumb subdomain (residues 682–697) [16].

Site-directed or random mutagenesis of DNA polymerase genes is an effective strategy for engineering modified enzymes with enhanced characteristics or novel properties for in vitro DNA manipulation [8,17,18,19]. A notable feature of archaeal family B DNA polymerases is their ability to recognize unrepaired uracil in the template DNA, which leads to replication stalling. This ability to bind deaminated bases is facilitated by a specialized “binding pocket” located in the N-terminal domain (involving residues 7, 36–37, 90–93, 111–116, 119, and 123) [20,21]. However, mutant forms of Pfu DNA polymerase, containing the Pro36His, Tyr37Phe, or Val93Gln substitutions, are well-characterized for their ability to bypass uracil in the template strand [20,21,22].

One of the widely used thermostable DNA polymerase, called Phusion, is a high-fidelity fusion enzyme. Phusion efficiently amplifies up to 10 kb DNA fragments with a rapid synthesis rate (0.5–1.5 kbp/min) and high-fidelity (error rate of 1 × 10^−6^–5 × 10^−6^) [3,17]. The engineered enzyme combines with the Pfu polymerase, containing a V93Q mutation to minimize uracil affinity [20,22], which is fused to the Sso7d protein from *Sulfolobus solfataricus* to boost processivity [8,23]. Creation of RT activity on base of Phusion enzyme one could expect to be a promising approach to generate thermostable RT. In the context of one-step RT-PCR, the presence of the Sso7d fusion domain could be an advantage as Sso7d is known to bind dsDNA, providing increased processivity and tolerance to amplification inhibitors, as was shown for Moloney murine leukemia virus RT [24].

Based on an analysis of the engineered RTs from B family DNA-dependent DNA polymerases [7,9,11,12,13,25] and a comparison of DNA polymerases from different microorganisms, we created six mutant forms of the Phusion enzyme in this study. These variants contain multiple amino acid substitutions in the palm domain and the 3′→5′ exonuclease domain. Enzymatic activity screening identified a variant, Pfu-M6, which possesses both DNA-dependent and RNA-dependent DNA polymerase activity. We have confirmed that Pfu-M6 is highly thermostable and retains its native DNA-dependent DNA polymerase function, and demonstrates the ability to catalyze reverse transcription followed by direct amplification of the resulting cDNA under standard PCR conditions.

## 2. Materials and Methods

### 2.1. Construction of Mutated Pfu DNA Polymerase Genes

Pfu V93Q-Sso7d DNA polymerase gene cloned into pET28c vector was kindly provided by professor D.O. Zharkov (ICHBFM SB RAS). Point mutations were introduced into the Pfu V93Q-Sso7d DNA polymerase gene by site-directed mutagenesis using a set of primers with Pfu DNA polymerase (SibEnzyme, Novosibirsk, Russia). All of the mutant plasmid DNA was synthesized by PCR amplification, and subsequently digested with the restriction enzyme MalI (SibEnzyme, Novosibirsk, Russia) to digest non-mutated parental methylated plasmid DNA. The mutated plasmids were transformed into *E. coli* Emax for nick repair. Introduced mutations were confirmed by sequencing each of the Pfu DNA polymerase mutant genes.

### 2.2. Expression and Purification of Pfu Mutant Forms

**Figure 1:**
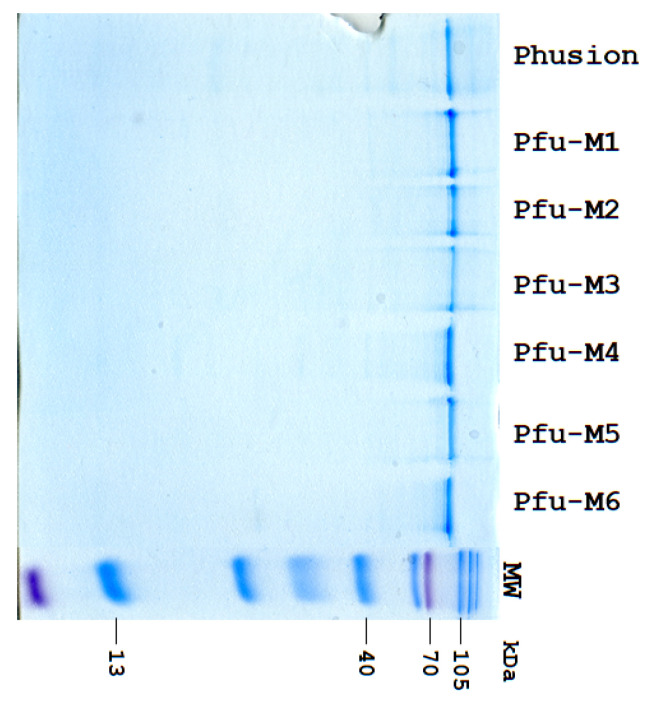
PAGE analysis of protein purity. Lanes: Phusion and Pfu-Mi (i = 1–6), M—Prestained Protein Marker VII (8–195 kDa). Original figures can be found in Supplementary Materials.

*Escherichia coli* strain Arctic (DE3) was transformed with pET28c-Pfu-Mi (i = 1 − 6). A starter culture (15 mL LB Broth with 50 mg/mL kanamycin) was grown overnight and used to inoculate a larger volume of LB Broth (1 L). When the cell density reached an OD_600_ of 0.6–0.8, protein expression was induced with IPTG (0.2 mM), and the culture was incubated for 20 h at 16 °C. The cells were then harvested, resuspended in 20 mM HEPES-KOH buffer (pH 7.8), 40 mM NaCl, and lysed using a French press. After removing the cellular debris by centrifugation (40,000× *g* for 40 min at 4 °C), the supernatant was subjected to a two-step purification process. First, the protein was purified using immobilized metal affinity chromatography (IMAC) on a Ni Sepharose resin (20 mM HEPES–NaOH pH 7.8, 500 mM NaCl, and 20 mM imidazole), leveraging a hexahistidine tag. The enzyme was eluted with a high-concentration imidazole buffer (20 mM HEPES–NaOH pH 7.8, 500 mM NaCl, and 600 mM imidazole). This was followed by heparin affinity chromatography, where the protein was eluted using a linear NaCl gradient (40 → 1000 mM). The purified polymerase fractions were collected, mixed with glycerol for cryoprotection, and stored at −20 °C. The final protein’s concentration and purity were assessed using the Bradford assay and SDS-PAGE, respectively (Figure 1).

### 2.3. DNA/RNA Substrates

Oligodeoxyribonucleotides were synthesized at BIOSSET LLC (Novosibirsk, Russia) and were not subjected to further purification/modification. Oligoribonucleotides were synthesized by PhD Novopashina D.S. in the Laboratory of RNA Chemistry of the Institute of Chemical Biology and Fundamental Medicine SB RAS. For each of the introduced amino acid substitutions, a pair of forward and reverse primers was used. To study the biochemical properties of the obtained mutant forms, 37-mer oligodeoxyribonucleotide DNAtemp37 and oligoribonucleotide RNAtemp37 were used as a template, and 22-mer oligodeoxyribonucleotide Pr-DNA-22 and oligoribonucleotide Pr-RNA-22 labeled with FAM were used as primers. To evaluate the activity of the obtained mutant forms under PCR conditions, the following oligonucleotides were used: TempDNA as DNA template of 90 nt length or TempRNA as RNA template of 90 nt length, and two DNA primers PrForw and PrRev of 20 nt length. The sequences of the oligodeoxyribonucleotides used in the work are given in Table 1.

### 2.4. Catalytic Activity of Mutant Forms of Pfu-Mi

To assess the enzymatic activity of the obtained mutant forms of Pfu-Mi, a set of substrates of the following composition was used: DNA template/DNA primer (D/D-substrate), DNA template/RNA primer (D/R-substrate), and RNA template/DNA primer (R/D-substrate) (Table 1). Stock solutions of substrates contained 50 μM of the corresponding template and primer. Annealing of the primers was carried out at 90 °C for two minutes, followed by slow cooling of the duplexes to 25 °C. The reaction mixtures contained 50 nM substrate, 0.5 μM enzyme, 1.25 mM dNTP or NTP in a buffer of 20 mM Tris-HCl, pH 8.8, 10 mM KCl, 10 mM (NH_4_)_2_SO_4_, 2 mM MgSO_4_, 0.1% Triton X-100. The reaction was carried out at 50 °C for 20 min. To stop the reaction, a stop solution (formaldehyde, 10 mM EDTA, 10 mM KOH, 2 μM complementary DNA template, xylene cyanol dye) was used. The results of the enzymatic reaction were analyzed by electrophoresis in 15% denaturing PAGE and visualized using a VersaDoc MP 4000 Molecular Digital Imaging System (Bio-Rad, Hercules, CA, USA).

### 2.5. Melting Temperature of the Mutant Form Pfu-M6

The melting temperature was determined using the differential scanning fluorimetry method. The experiment was carried out in a QuantStudio 5 amplifier (Applied Biosystems, Life Technologies, Carlsbad, CA, USA). ProteOrange Protein Gel Stain, 5000× (OOO “Lumiprob RUS”, Rostov-on-Don, Russia) with a maximum absorption wavelength of 470 nm and an emission wavelength of 570 nm was used as a fluorescent dye. The melting point of the enzymes (8 μM) was measured in a buffer solution of 40 mM HEPES-KOH, 400 mM NaCl, 2 M guanidinium chloride by heating the samples from 25 °C to 99.9 °C at a rate of 0.015 °C/s.

### 2.6. Kinetic Parameters of the Polymerase Reaction

To determine the kinetic parameters of DNA-dependent DNA polymerase activity, a duplex of the DNA template with a DNA primer (D/D-substrate) was used as a substrate. The reaction mixture contained 50 nM of the prepared substrate, 0.5 μM of the enzyme, and a mixture of four dNTPs at concentrations of 5, 10, 20, 50, 100, 200, μM in a buffer of 20 mM Tris-HCl, pH 8.8, 10 mM KCl, 10 mM (NH_4_)_2_SO_4_, 2 mM MgSO_4_, 0.1% Triton X-100. The reaction was carried out at 37 °C and stopped with stop solution at certain time intervals: 10 s, 20 s, 30 s, 45 s, 1 min, 5 min, 10 min, and 20 min. Determination of the kinetic parameters of the reverse transcription reaction (RNA-dependent DNA polymerase activity) was carried out similarly, using a duplex of RNA template with DNA primer (R/D-substrate) as a substrate. The dNTP concentrations were 50, 100, 200, 500, 750, and 1000 μM, and time intervals were 45 s, 1 min, 3 min, 5 min, 10 min, 20 min, 30 min, and 40 min. The results of the enzymatic reactions were analyzed by electrophoresis in 15% denaturing PAGE, visualized using a gel-documenting system VersaDoc MP 4000 Molecular Digital Imaging System (Bio-Rad, Hercules, CA, USA). The obtained electropherograms were processed using Gel Analyzer software, v.4.0 (Media Cybernetics, Rockville, MD, USA). The kinetic curves of the reaction products’ accumulation were used to calculate the observed rate constants k^obs^ of dNTP addition using the exponential equation $y=A_{0}\left(1-e^{-kobs\cdot t}\right)$. The dependences of the observed rate constant of the polymerase reaction kobs on the dNTP concentration were approximated using the equation $k^{\mathrm{obs}}=\frac{k_{\mathrm{cat}}\left[\mathrm{dNTP}\right]}{K_{m}+\left[\mathrm{dNTP}\right]}$.

### 2.7. Testing of PCR Activity

To test the enzyme activity under PCR conditions, 90-mer DNA or RNA oligonucleotides were used as a template, as well as forward and reverse DNA primers (Table 1). To analyze DNA-dependent DNA polymerase activity, a DNA template, forward and reverse DNA primers, and a dNTP mixture were used. To test RT activity, an RNA template, forward and reverse DNA primers, and a dNTP mixture were used. The reaction mixture contained 40 ng of DNA/RNA template, 1.25 mM dNTP, 0.5 μM forward and reverse DNA primer, and 30 nM of the tested DNA polymerase in a buffer of 20 mM Tris-HCl, pH 8.8, 10 mM KCl, 10 mM (NH_4_)_2_SO_4_, 2 mM MgSO_4_, and 0.1% Triton X-100. PCR was performed in a QuantStudio 5 amplifier (Applied Biosystems, Life Technologies, USA). The reaction conditions were as follows: one initial denaturation step (94 °C, 4 min) followed by 5–15 cycles of amplification (94 °C, 30 s; 55 °C, 30 s; 72 °C, 30 s) and a final 2 min extension at 72 °C. The results of DNA and RNA template amplification under PCR conditions were analyzed using gel electrophoresis in 2.5% agarose gel in the presence of ethidium bromide.

## 3. Results and Discussion

### 3.1. Selection of Amino Acid Residues Potentially Influencing the Emergence of RT Activity

The development of family B DNA polymerases for high-fidelity DNA amplification by PCR, synthesis of modified unnatural nucleic acids known as xeno-nucleic acids (XNAs), and creation of reverse transcriptases which convert RNA to DNA have been researched extensively over recent decades [7]. The family B DNA polymerases have a well-known structure resembling a right hand with three main subdomains: the “palm,” “fingers,” and “thumb” in the polymerase domain and individual 3′-5′-exonuclease domain, which is located in the N-terminus of the enzyme [26].

Two of the well-studied family B DNA polymerases with altered substrate specificity are Tgo from *Thermococcus gorgonarius* [27] and KOD from *Thermococcus kodakarensis* [28]. Both enzymes were used for the creation of multiple mutant forms for efficient synthesis of various XNAs [7]; moreover, for KOD enzyme, it was described an RTX-variant, which simultaneously possesses RNA- and DNA-dependent DNA polymerase activities [5,10]. Numerous engineered variants from Tgo, capable of performing the efficient synthesis of various XNAs and reverse DNA synthesis on an XNA strand, were described [9,11,12,25,29].

**Figure 2:**
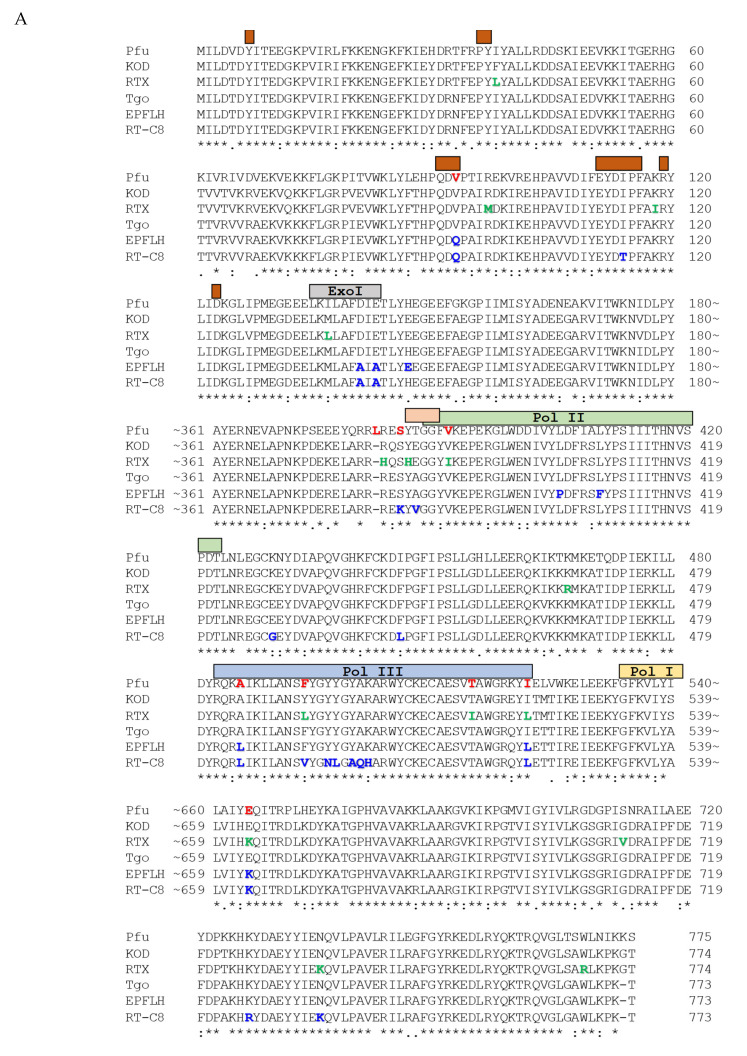

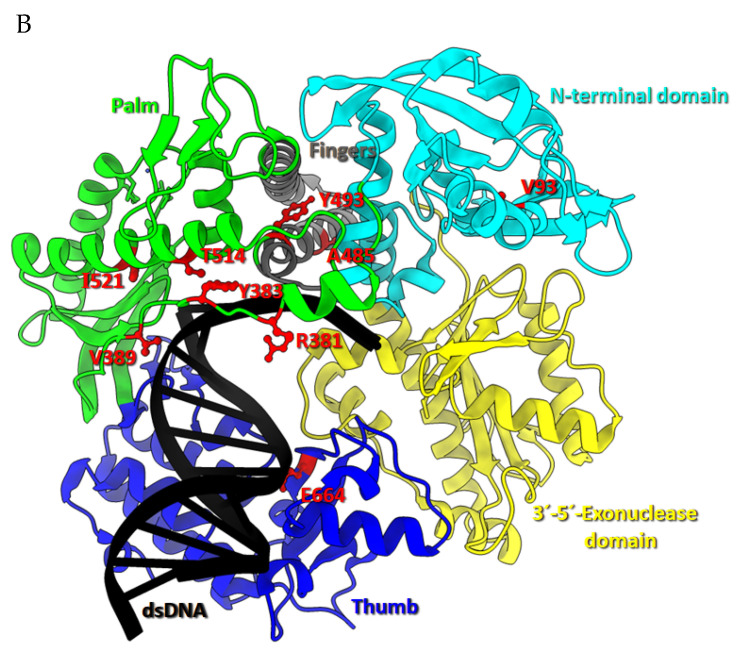
(**A**) Sequence alignment of Pfu, KOD, RTX [10], Tgo, EPFLH [25], and RT-C8 [9] enzymes. The amino acid residues of the KOD sequence whose replacement led to the appearance of RT properties in RTX-variant are colored in green; for Tgo mutant forms, they are colored in blue. The selected for substitution residues for Pfu are colored in red. The symbols are shown below each line of the alignment, denoting the degree of conservation observed in each column: “*” indicates fully conserved residues, “:” indicates conservation between groups of strongly similar properties, “.” indicates semi-conservative residues. Conserved regions of family B DNA polymerases are highlighted: Pol I through Pol III—yellow, green and blue rectangles, respectively; Exo I is gray rectangle; U-binding pocket including amino acid residues 7; 36–37; 90–93; 111–116; 119; 123 is highlighted by brown rectangles; Y-G(G/A) DNA-binding motif is highlighted by beige rectangle. (**B**) Structure of binary complex of DNA polymerase KOD from *Thermococcus kodakarensis* with dsDNA (Protein Data Bank [PDB] ID: 4K8Z) with marking amino acid residues selected for substitutions. The single polypeptide chain of 775 amino acids is folded into five distinct structural domains: the N-terminal domain (cyan, amino acid residues 1–130, 327–368), the 3′→5′ exonuclease domain (yellow, amino acid residues 131–326), the palm domain (green, amino acid residues 369–450 and 501–588), the fingers (gray, amino acid residues 451–500), and the thumb region (blue, amino acid residues 589–775). DNA is highlighted in black. The side chains of the residues selected for substitutions (V93Q, L381H, S384H, V390I, A486L, F494L, T515I, I522L, and E665K) are shown with atoms represented as red ball sticks.

To select amino acid residues of Pfu DNA polymerase, which potentially could affect selectivity to RNA template, we have aligned sequences of Pfu, KOD and Tgo enzymes (Figure 2A). Several regions are distinguished in the polypeptide chain of 775 amino acids: Pol I-Pol VI, the conserved regions of family B DNA polymerases and Exo I-Exo III, conserved motifs of the 3′-5′-exonuclease domain. In the alignment, the wild-type KOD, Tgo, and Pfu sequences and engineered variants RTX [10], EPFLH [25], and RT-C8 [9] are shown. The amino acid residues of the KOD sequence whose replacement led to the appearance of RT properties in RTX-variant (green) are highlighted. It is interesting to note that in the Tgo sequence, the amino acid residues whose replacement led to the appearance of mutant forms capable of synthesizing DNA along the XNA chain (blue) are not the same as with the KOD sequence, but typically located in the same critical regions (Figure 2A). Sequence alignment of Pfu, KOD, and Tgo enzymes allow us to select nine amino acid residues in the Pfu enzyme for substitutions (highlighted in red in Figure 2A), which can potentially lead to the emergence of RT activity: V93Q, L381H, S384H, V390I, A486L, F494L, T515I, I522L, and E665K (Table 2). Mapped on the structure of KOD-DNA complex, selected for substitution residues are clustered at the region of enzyme interaction with primer–template duplex (Figure 2B). As mentioned above, Val93Gln mutation minimizes the uracil affinity. Amino acids at 381, 384, and 389 (390 in Pfu) positions are located near the template strand and their mutations probably resulted in reducing template binding [5]. The substitution of Ala485 residue (Ala486 in Pfu) probably contributes to the rotation of the finger domain changing the geometry of the enzyme active site, providing an enhanced incorporation of unnatural dNTP. The importance of Ile521Leu substitution for the function of reverse transcription from XNA into DNA was shown by Pinheiro V.B. et al. [12]. Residue Glu664 is one of the steric gates of DNA polymerase and contacts DNA through coordinated water molecules in the minor groove of the DNA helix. Its substitution is necessary for efficient XNA/RNA synthesis [30]. The expected effects of selected amino acid substitutions are summarized in Table 2.

Combination of these substitutions leads to creation of six mutant forms Pfu-Mi for analysis of enzyme properties (Table 3).

### 3.2. Evaluation of the Enzymatic Activity of Mutant Forms of Pfu-Mi

**Figure 3:**
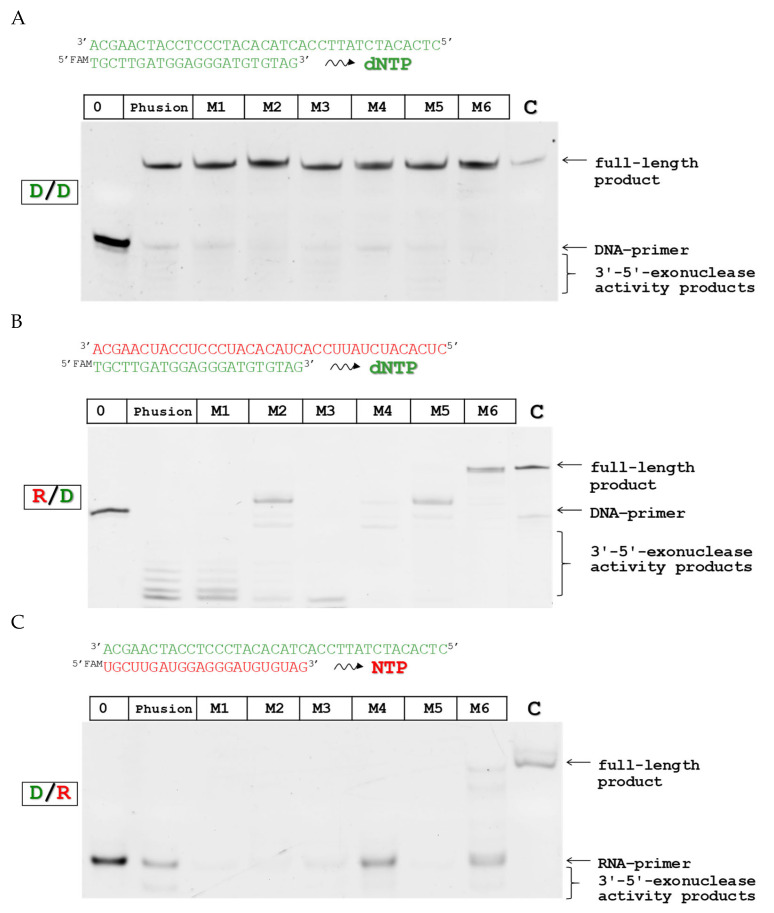
Catalytic activity of mutant forms of Pfu-Mi: (**A**) DNA-dependent DNA polymerase, (**B**) RNA-dependent DNA polymerase, and (**C**) DNA-dependent RNA polymerase. Electropherograms of 15% denaturing PAAG after electrophoretic separation of the polymerase reaction products. Lanes’ designations: “0” represents initial substrate without any treatment; “Phusion” enzyme was used in the reaction as control, “Pfu-M1”-“Pfu-M6” activity of created mutant forms, “C” represents control of the full-size product. The color code for primer and template chains is used: green for DNA and red for RNA. Original figures can be found in Supplementary Materials.

To evaluate the catalytic activity of the obtained mutant forms, a polymerase reaction was carried out using a 37-mer template and a 22-mer FAM-labeled primer (Table 1). Based on these oligonucleotides, four types of substrates were prepared containing DNA template/DNA primer (D/D-substrate), DNA template/RNA primer (D/R-substrate), and RNA template/DNA primer (R/D-substrate), which made it possible to establish the ability of the enzymes to extend the primer chain by adding ribo- or 2′-deoxyribonucleotides (Figure 3).

Analysis of the enzymatic activity of the obtained mutant forms showed that all enzymes retained the activity of DNA-dependent DNA polymerase (Figure 3A). At the same time, the forms Pfu-M2, Pfu-M5, and Pfu-M6 have RNA-dependent DNA polymerase activity (Figure 3B), although the full-length 37-mer product was detected only in the case of Pfu-M6. For Pfu-M2 and Pfu-M5 mutant forms, fragments in the gel show only very short incorporations. Since all variants being tested retain their exonuclease activity, it is likely that these variants are in balance between exonuclease and polymerase activities unlike Pfu-M6.

It was shown that Pfu-M6 demonstrated, in addition, the weak activity of DNA-dependent RNA polymerase, with a full-length product visible on a gel (Figure 3C). However, the RNA synthesis was ineffective in comparison with corresponding RNA-dependent DNA synthesis, given that over half of the primer is not extended (Figure 3B). Similar results have been reported for Tgo-based RNA polymerases as well [30]. It was demonstrated for DNA polymerase 9°N from the *Thermococcus* species that mutant form Leu408Gln can perform RNA synthesis on a DNA template [31]. In the wild-type enzyme, the residue Leu408 apparently participates in substrate discrimination. In addition, the neighboring residue Tyr409 prevents rNTP incorporation through a steric conflict with the 2′-OH group. The substitutions of these residues was shown to lead to the emergence of RNA polymerase activity [12,13,30]. The further improvement of Pfu-M6 in this direction can provide us with an enzyme with DNA/RNA-dependent RNA/DNA polymerase activity.

Thus, among the obtained variants, Pfu-M6 showed the highest efficiency as an RT (RNA-dependent DNA polymerase activity). In this regard, in the next stages of the work, the enzymatic characteristics of only the Pfu-M6 variant were determined in more depth.

### 3.3. Melting Temperature

**Figure 4:**
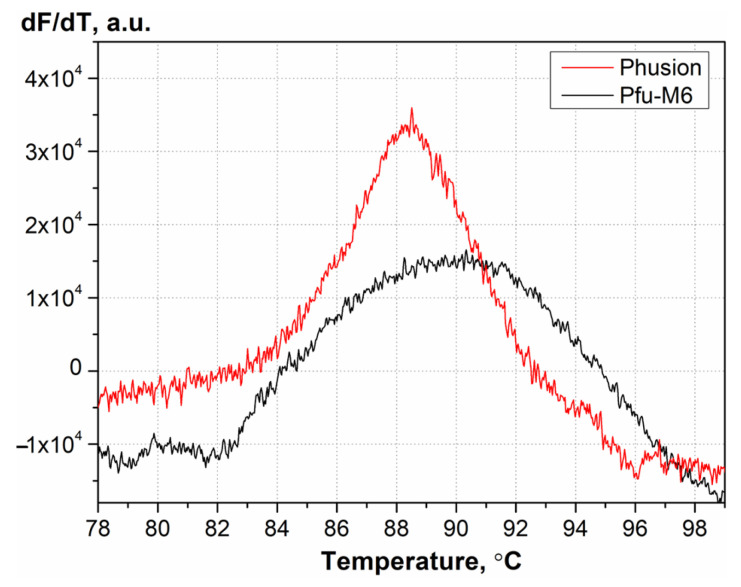
Melting curves of Phusion and Pfu-M6 enzymes in the presence of 2 M guanidinium and the fluorescent dye ProteOrange Protein Gel Stain.

The thermal stability of the enzymes, indicated by their melting temperature (T_m_), was determined by Differential Scanning Fluorimetry (DSF). This technique monitors the fluorescence of a dye that binds to hydrophobic protein regions exposed during thermal denaturation [32,33]. Because the wild-type Pfu polymerase is extremely stable and does not fully denature at 100 °C, guanidinium hydrochloride was added to facilitate unfolding [32]. The first derivative curves of melting profile are shown in Figure 4. Analysis of the melting profiles revealed that the mutant Pfu-M6 had a T_m_ of 88.8 ± 0.4 °C, which is not significantly different from the original Phusion enzyme’s T_m_ of 88.4 ± 0.3 °C under the same conditions.

### 3.4. Kinetic Parameters of Substrate Extension

**Figure 5:**
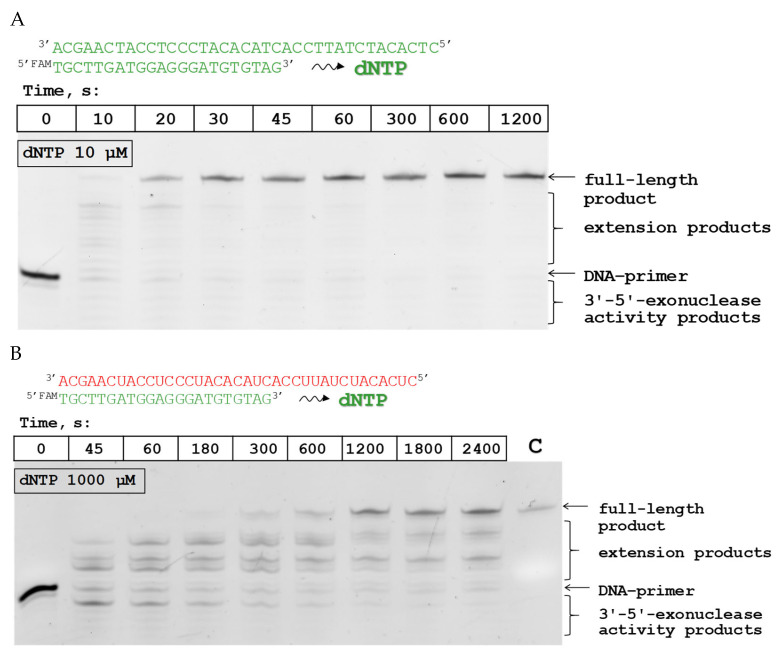
DNA primer elongation under the interaction of Pfu-M6 with the D/D- (**A**) and R/D-substrates (**B**) at 37 °C. Reaction conditions are given in Section 2. “C” represents control of the full-size product (37-mer oligoribonucleotide RNAtemp37_Contr). The color code for primer and template chains is used: green for DNA and red for RNA. Original figures can be found in Supplementary Materials.

The kinetic characteristics of dNTP addition by Pfu-M6 during DNA-dependent DNA polymerase activity and RT activity were determined at 37 °C. The employment of the kinetic parameters at 37 °C allowed us to evaluate the overall impact of amino acid substitutions on the DNA polymerase properties. For this purpose, DNA primer extension was performed in the composition of D/D and R/D substrates (Table 1) in the presence of an equimolar mixture of dNTP. Figure 5 shows examples of gel electrophoretic separation of DNA primer extension products in the composition of D/D and R/D substrates.

**Figure 6:**
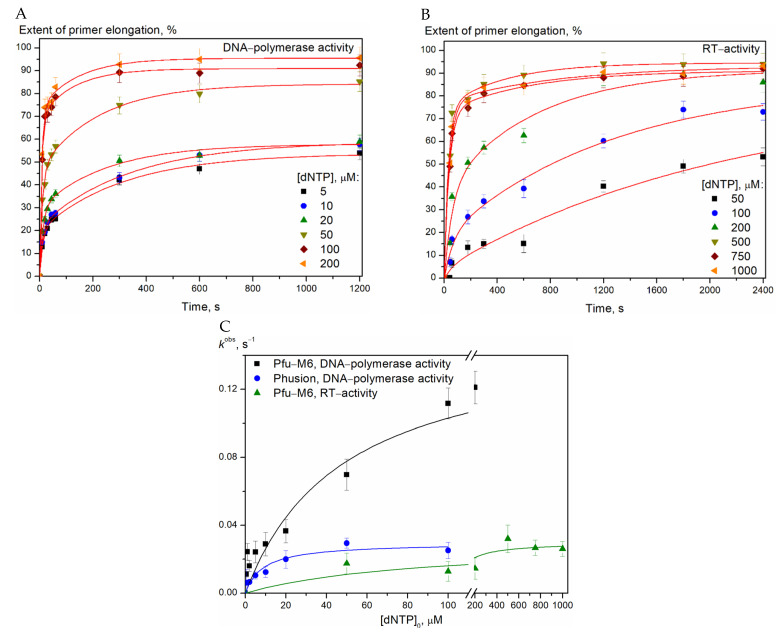
Kinetic curves of the DNA primer extension under the interaction of Pfu-M6 with the D/D- (**A**) and R/D-substrates (**B**) at 37 °C. Dependence of the observed rate constant k^obs^ on the dNTP concentration (**C**).

The kinetic curves of the accumulation of the enzymatic reaction products at different dNTP concentrations (Figure 6) were approximated using an exponential equation, which allowed us to calculate the observed primer extension rate constants. The dependence of *k*^obs^ on the dNTP concentration was approximated using the Michaelis equation; the obtained values of the Michaelis constants and the catalytic rate constant are presented in Table 4.

Although steady-state kinetic parameters do not reveal the specific effects resulting from amino acid substitution, they indicate overall enzyme function [34,35]. We observed a 5-fold difference in *K*_m_ and *k*_cat_ when comparing Phusion to the Pfu-M6. The *k*_cat_/*K*_m_ ratio for DNA-dependent DNA polymerase activity was similar for Phusion and Pfu-M6. In the case of Pfu–M6, moving from a DNA template to an RNA template, the value of the *k*_cat_ parameter decreased by five folds, and the value of the *k*_cat_/*K*_m_ ratio decreased by ten folds. Kinetic analyses assumed that Pfu–M6 uses RNA templates with lower efficiencies than DNA templates by lowering both *K*_m_ and *k*_cat_ constants on RNA:DNA heteroduplexes. This indicates a lower efficiency of RNA-depended DNA synthesis compared with DNA-dependent DNA synthesis. The decrease in *k*_cat_/*K*_m_ ratio was described for RTX-variant of KOD enzyme on RNA templates as well [10].

### 3.5. Pfu-M6 Activity Under PCR Conditions

**Figure 7:**
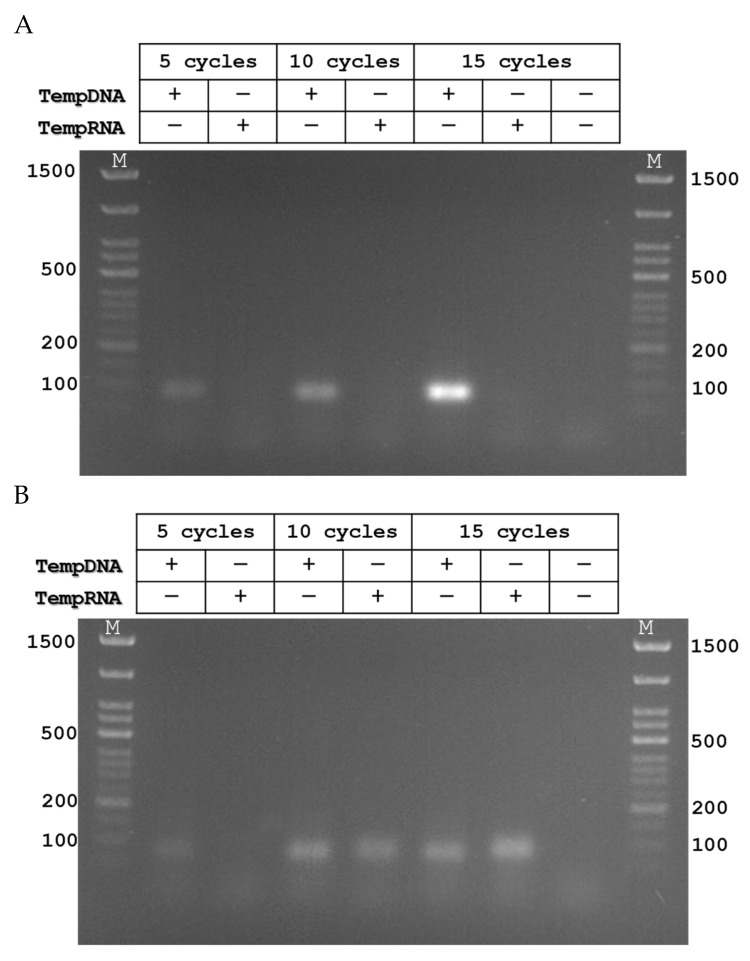
Catalytic activity of Pfu-M6 under PCR conditions. (**A**) Amplification of TempDNA/TempRNA template with dNTP mix by Phusion. (**B**) Amplification of TempDNA/TempRNA template with dNTP mix by Phu-M6. PCR products were analyzed using 2% agarose gel electrophoresis. Lane M contains DNA molecular size markers (p Step50 plus, Biolabmix, Novosibirsk, Russia). The cycling protocol consisted of one initial denaturation step at 94 °C for 3 min followed by 5/10/15 cycles of 94 °C for 15 s, 50 °C for 15 s, and 72 °C for 15 s. The amount of cycles is indicated along the top of the figure. Original figures can be found in Supplementary Materials.

To test the activity of Pfu-M6 under PCR conditions, model oligonucleotides were used: DNA and RNA templates of 90 nt in length and DNA primers of 20 nt in length (Table 1). This set of oligonucleotides made it possible to test the presence of DNA-dependent DNA polymerase activity, RNA-dependent RNA polymerase activity, RNA-dependent DNA polymerase activity, and DNA-dependent RNA polymerase activity. It was found that Pfu-M6 retains DNA-dependent DNA polymerase activity and also carries out a reverse transcription with subsequent amplification of the target cDNA under PCR conditions (Figure 7).

Using modified next-generation sequencing (NGS), it was shown that the balance between fidelity and reaction efficiency in RT could be achieved by the optimized PCR condition [36,37]. For RTX [36,38], the reaction efficiency of cDNA synthesis was more affected by MgCl_2_ and dNTP concentrations in comparison with HIV-1 RT [36,39], thermostable variant of Moloney murine leukemia virus RT [36,40], and DNA polymerase K4polL329A from *Thermotoga petrophila* K4 with RT activity [36,41]. As we used a standard protocol for Pfu enzyme, one can expect that optimization of reaction condition could increase fidelity of enzyme action. Keeping in mind that Pfu-M6 retains proofreading activity and the tested mutations were deliberately engineered to maintain error-proofing, we could propose that the fidelity of cDNA synthesis should be maintained at a high level. The quantifying of the fidelity of the cDNA synthesis by NGS will be performed in our future research.

## 4. Conclusions

Directed enzyme engineering is widely used to improve the properties of DNA polymerases. The literature describes various mutant forms of family B DNA polymerases containing substitutions within their dNTP-binding sites, DNA-binding sites, and individual structural domains. These substitutions alter enzymatic properties and can confer the ability to synthesize DNA using an RNA or even XNA template.

In this work, we analyzed sequence and selected nine amino acid residues within the DNA-dependent DNA polymerase from *Pyrococcus furiosus* (Pfu). Replacing these residues could potentially confer RNA-dependent DNA polymerase activity (reverse transcriptase properties) on the enzyme. We constructed six expression vectors encoding mutant forms of the Pfu enzyme, each containing a distinct combination of substitutions at the selected residues.

Under isothermal conditions, only one of the six mutant forms, designated Pfu-M6, which contains eight amino acid substitutions, retained DNA-dependent DNA polymerase activity. Notably, this variant also exhibited RNA-dependent DNA polymerase activity (reverse transcriptase).

When assessed under standard PCR conditions Pfu-M6 demonstrated the ability to amplify DNA from both DNA and RNA templates. The template indifference in DNA synthesis makes Pfu-M6 a promising variant for molecular biology applications requiring the generation of DNA copies from RNA templates in the first PCR cycles with subsequent simultaneous dual amplification of both RNA and DNA templates.

## Figures and Tables

**Table 1:** Sequences of the oligonucleotides used in the work.

Shorthand *	Sequence
**D**/**D**-substrate	3′-d(ACGAACTACCTCCCTACACATCACCTTATCTACACTC)-5′ 5′-FAM-d(TGCTTGATGGAGGGATGTGTAG)-3′
**R**/**D**-substrate	3′-ACGAACUACCUCCCUACACAUCACCUUAUCUACACUC-5′ 5′-FAM-d(TGCTTGATGGAGGGATGTGTAG)-3′
**D**/**R**-substrate	3′-d(ACGAACTACCTCCCTACACATCACCTTATCTACACTC)-5′ 5′FAM-UGCUUGAUGGAGGGAUGUGUAG-3′
TempDNA	d(AGCCAATTTGGTATTCTTAACTGCTATAAGTGTGTTTAAGGCTAGTCCGTTATCAACTTGAAAAAGTGGCACCGAGTCGGTGCTTTTTTT)
TempRNA	r(AGCCAAUUUGGUAUUCUUAACUGCUAUAAGUGTGUUUAAGGCUAGUCCGUUAUCAACUUGAAAAAGUGGCACCGAGUCGGUGCUUUUUUU)
PrForw	d(AGCCAATTTGGTATTCTTAA)
PrRev	d(AAGCACCGACTCGGTGCCAC)

* For D/D, R/D and D/R-substrates the color code for primer and template chains is used: green for DNA and red for RNA.

**Table 2:** Location and expected effect of selected amino acid substitutions.

Substitution	Location	Expected Effect
V93Q	N-terminal domain	The Val93 residue is located in a hydrophobic α-helix (amino acid residues 90–97) that forms a side of the U-binding pocket. The V93Q substitution reduces the enzyme’s affinity for uracil-containing DNA and dUTP [20,22].
L381H	Junction of Palm and 3′-5′ exonuclease domains	Both residues are located near the template chain and presumably lead to weakening of the binding to the template.
S384H		
V390I	Junction of Palm and 3′-5′ exonuclease domains	Located near the template strand, presumably results in steric repulsion of the template strand.
A486L	Fingers	Presumably results in steric repulsion between the finger domain and the N-terminal domain, increasing plasticity.
F494L	Fingers	Presumably reduces steric contacts in the finger domain, increasing plasticity.
T515I	Palm	Located near the active site, presumably reduces steric contacts, increasing plasticity.
I522L		
E665K	Thumb	Presumably increases the efficiency of binding to the newly synthesized DNA strand.

**Table 3:** Created mutant forms of Pfu polymerase.

Enzyme	Substitutions
Pfu-M1	V93Q, T515I, I522L, E665K
Pfu-M2	V93Q, F494L, I522L, E665K
Pfu-M3	V93Q, L381H, S384H, V390I, T515I, I522L, E665K
Pfu-M4	V93Q, L381H, S384H, V390I, F494L, I522L, E665K
Pfu-M5	V93Q, L381H, S384H, V390I, A486L, T515I, I522L, E665K
Pfu-M6	V93Q, L381H, S384H, V390I, A486L, F494L, I522L, E665K

**Table 4:** Kinetic parameters of DNA- and RNA-dependent DNA polymerase activity of Phusion and Pfu-M6 (37 °C).

Enzyme	DNA-Dependent DNA Polymerase Activity			RNA-Dependent DNA Polymerase (RT) Activity		
	*k*_cat_, s^−1^	*K*_m_, µM	*k*_cat_/*K*_m_ (µM^−1^ × s^−1^)	*k*_cat_, s^−1^	*K*_m_, µM	*k*_cat_/*K*_m_ (µM^−1^ × s^−1^)
Phusion	0.03 ± 0.003	8.8 ± 1.7	0.0034	NA *		
Pfu-M6	0.15 ± 0.02	48 ± 17	0.0031	0.03 ± 0.003	86 ± 26	0.00035

* NA, not applicable.

## Data Availability

The original contributions presented in this study are included in the article/Supplementary Materials. Further inquiries can be directed to the corresponding author.

## Supplementary Materials

The following supporting information can be downloaded at: https://www.mdpi.com/article/10.3390/biom15111507/s1, File S1: PAGE, AG, SDS-PAGE original images.
