# Supplementary figures and images for: Engineering a Thermostable Reverse Transcriptase for RT-PCR Through Rational Design of Pyrococcus furiosus DNA Polymerase
Source: Biomolecules. 2025 Oct 24;15(11):1507. doi: 10.3390/biom15111507 (PMC12650659; doi:10.3390/biom15111507)

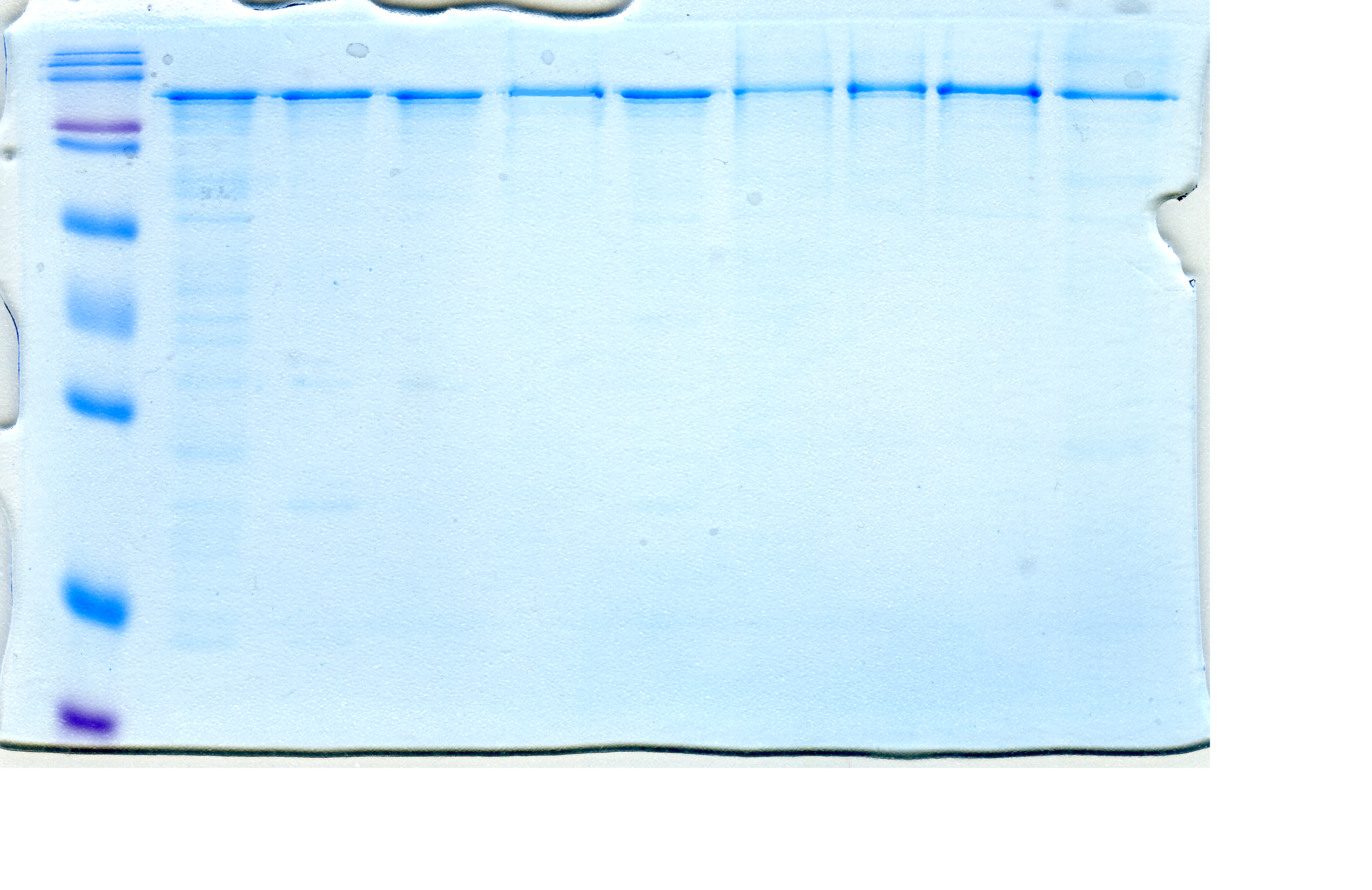

Supplement: Supplementary file 1 [file biomolecules-15-01507-s001.zip › Fig. 1.tif]

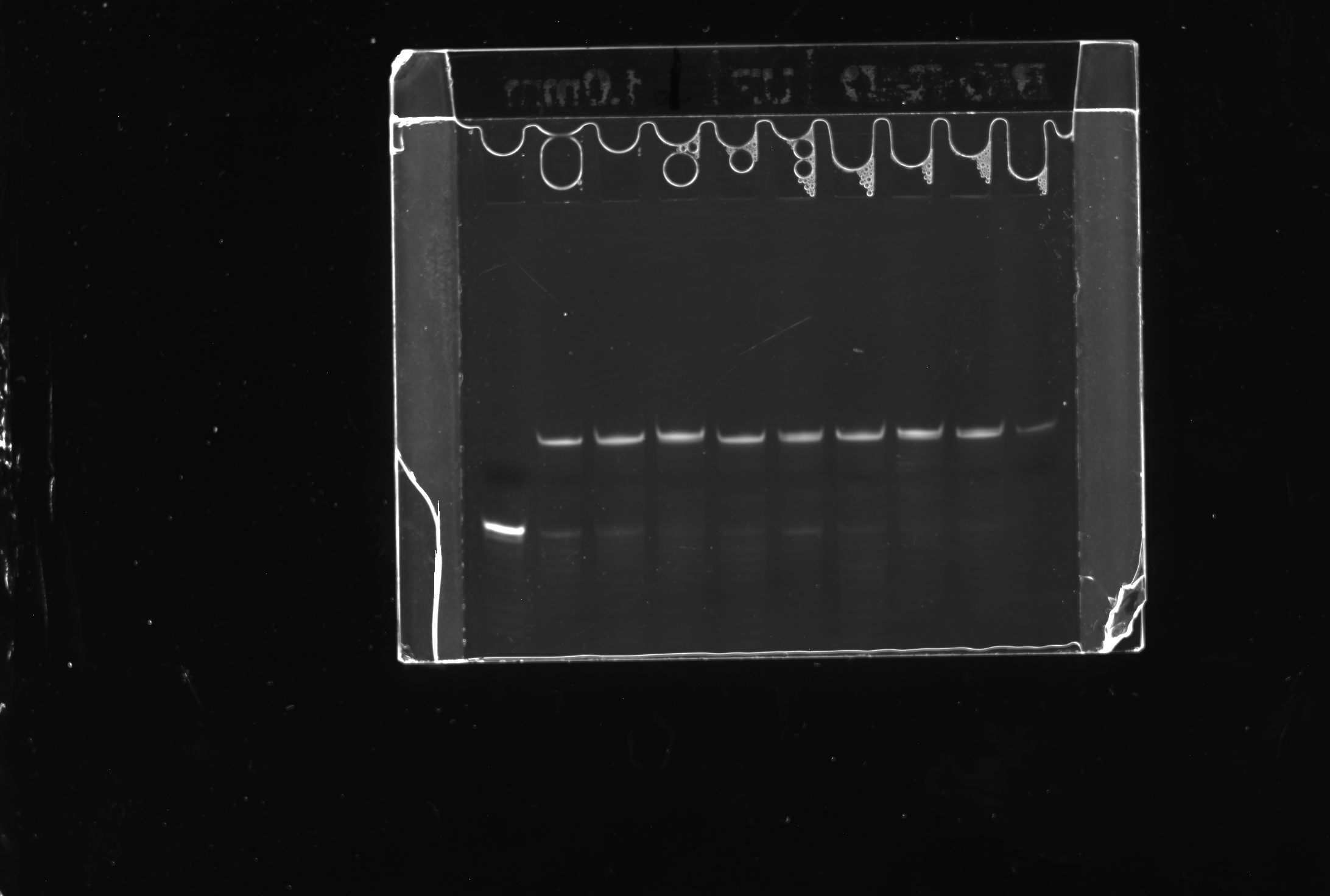

Supplement: Supplementary file 1 [file biomolecules-15-01507-s001.zip › Fig. 3_D-D.tif]

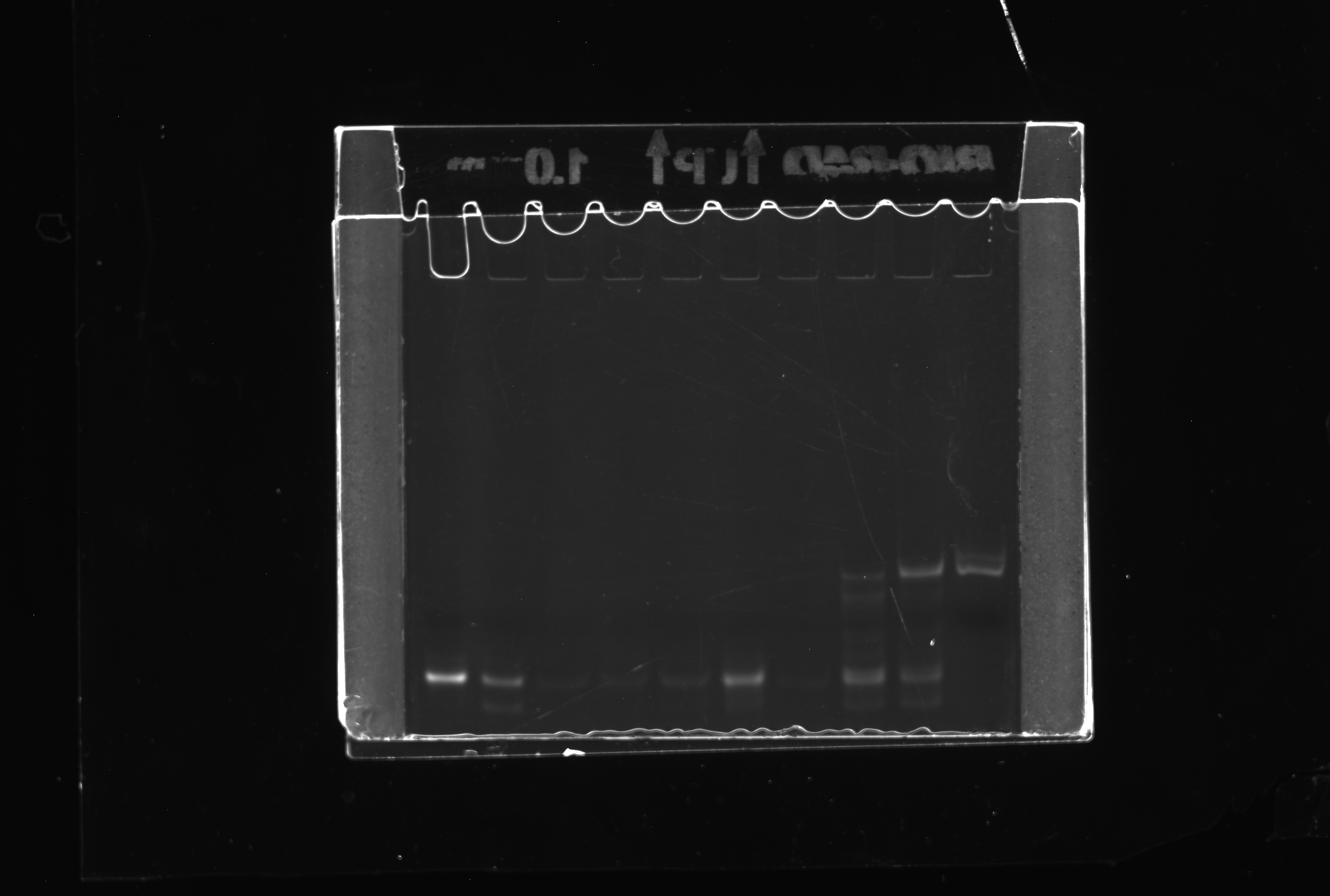

Supplement: Supplementary file 1 [file biomolecules-15-01507-s001.zip › Fig. 3_D-R.tif]

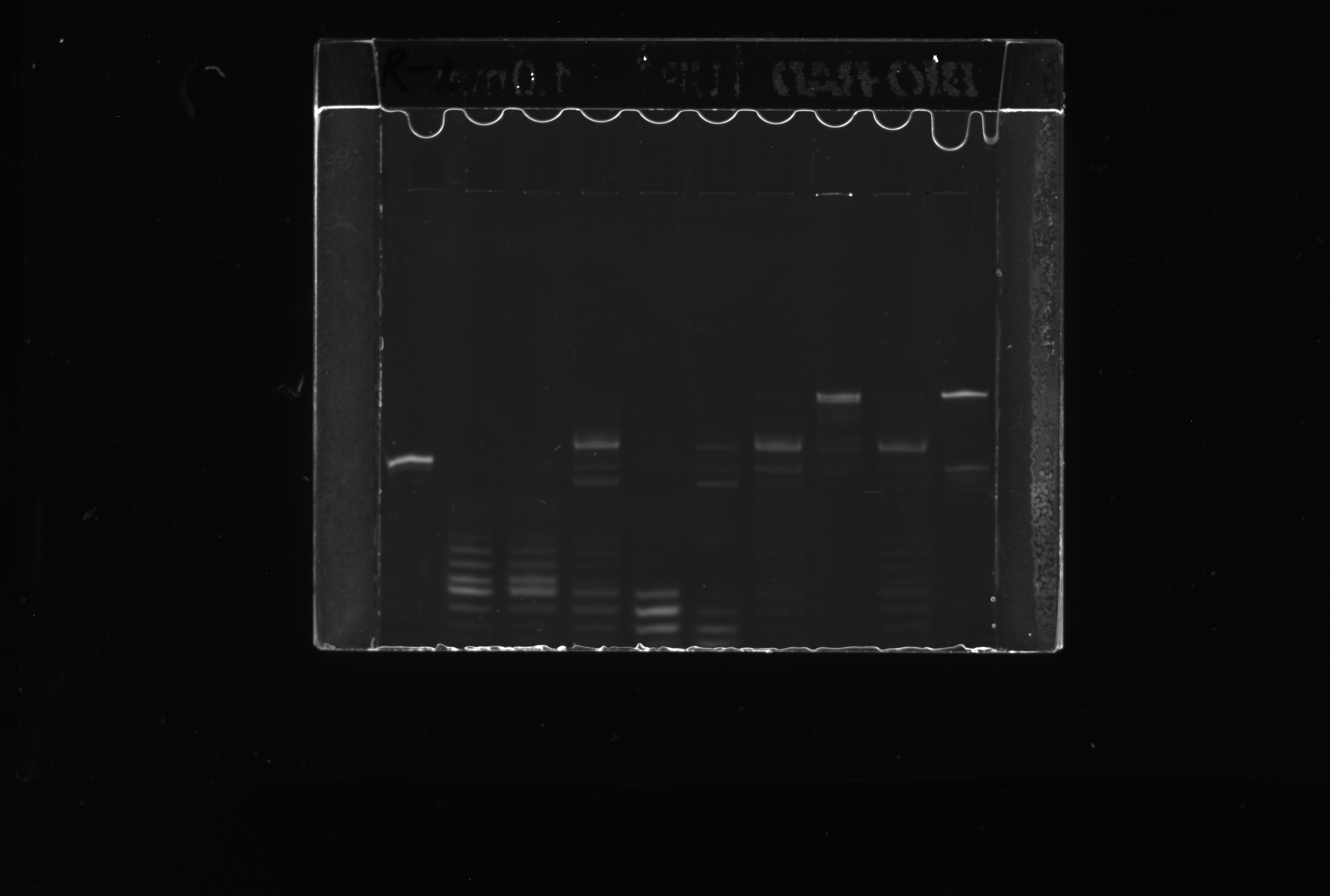

Supplement: Supplementary file 1 [file biomolecules-15-01507-s001.zip › Fig. 3_R-D.tif]

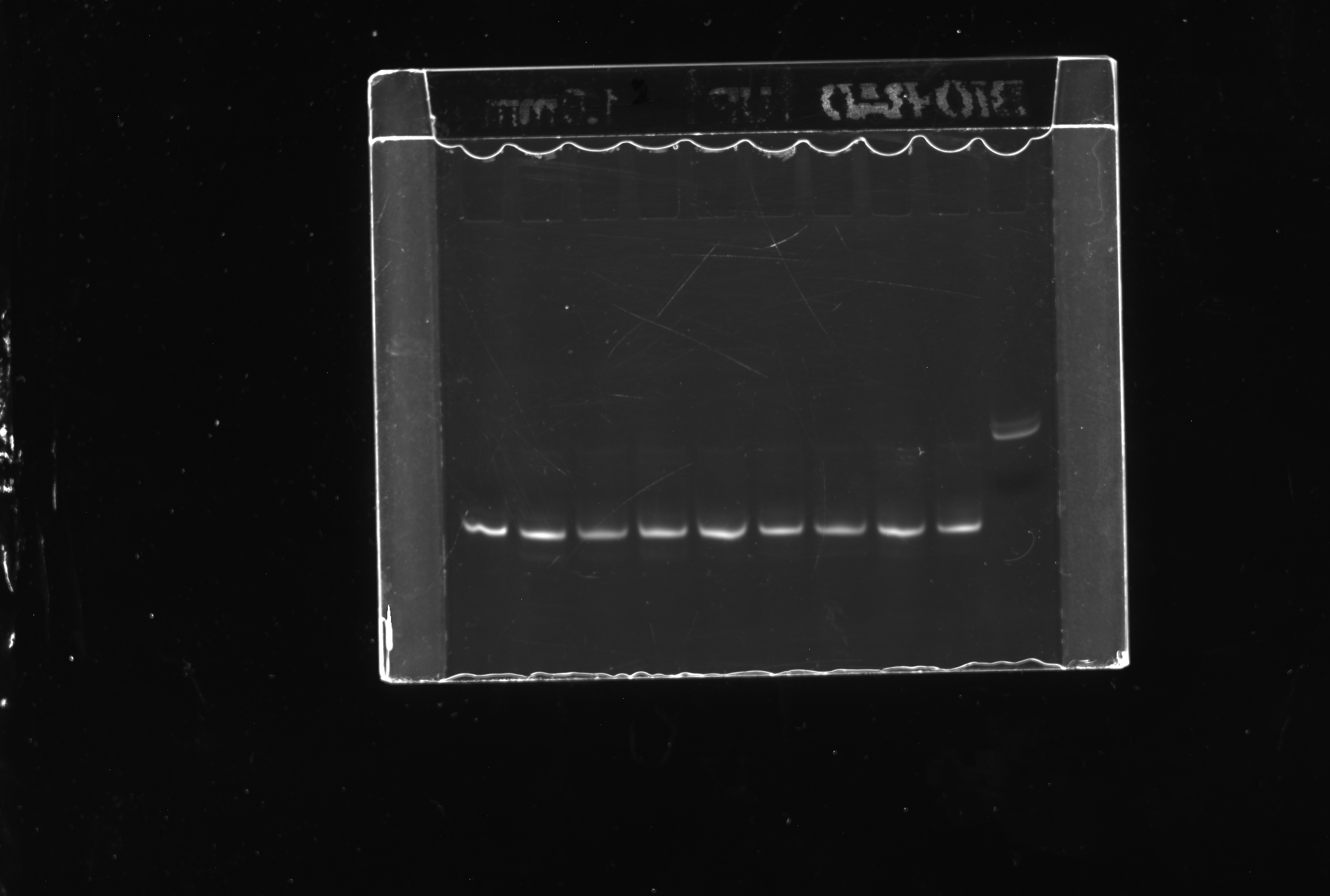

Supplement: Supplementary file 1 [file biomolecules-15-01507-s001.zip › Fig. 3_R-R.tif]

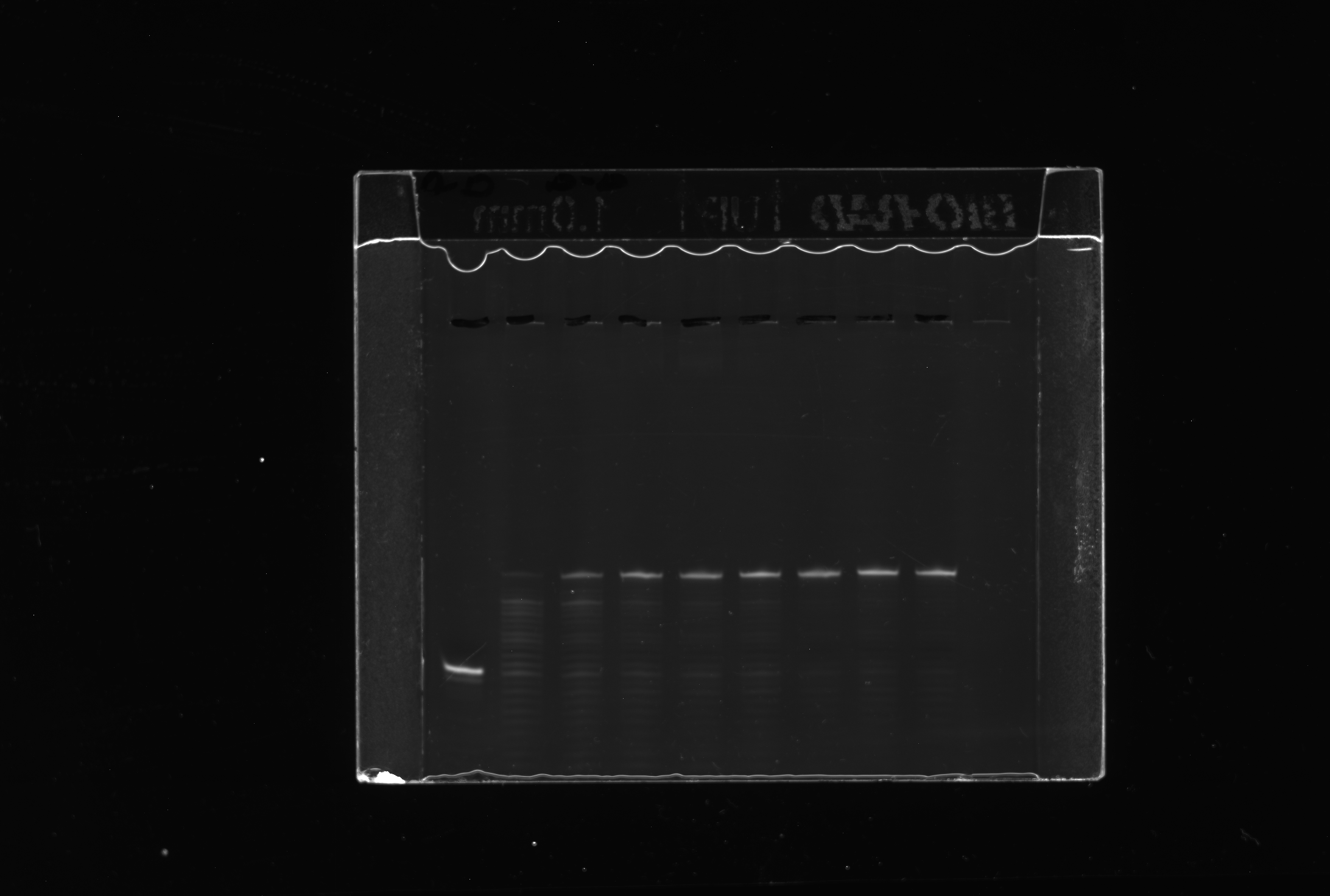

Supplement: Supplementary file 1 [file biomolecules-15-01507-s001.zip › Fig. 5_D-D.tif]

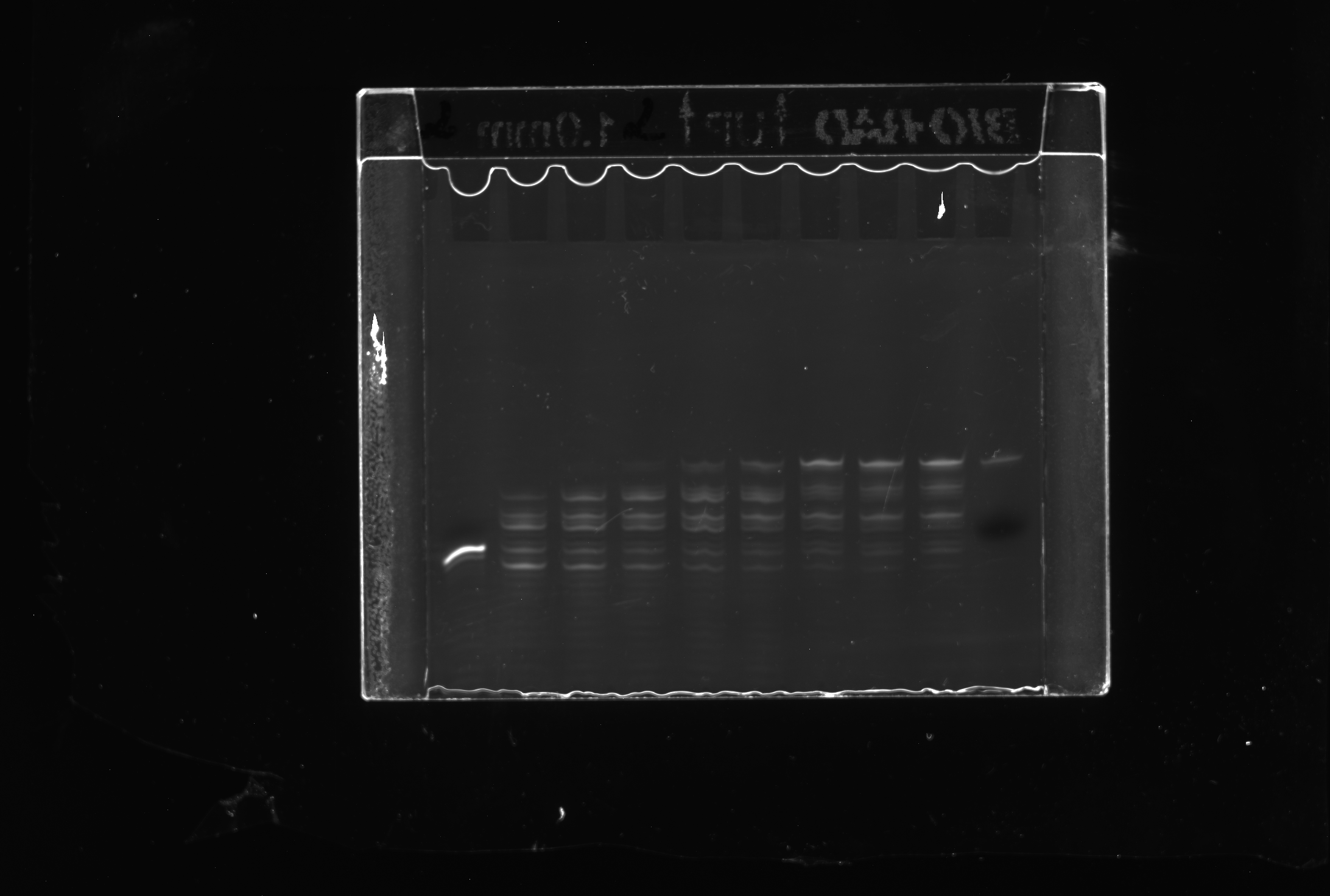

Supplement: Supplementary file 1 [file biomolecules-15-01507-s001.zip › Fig. 5_R-D.tif]

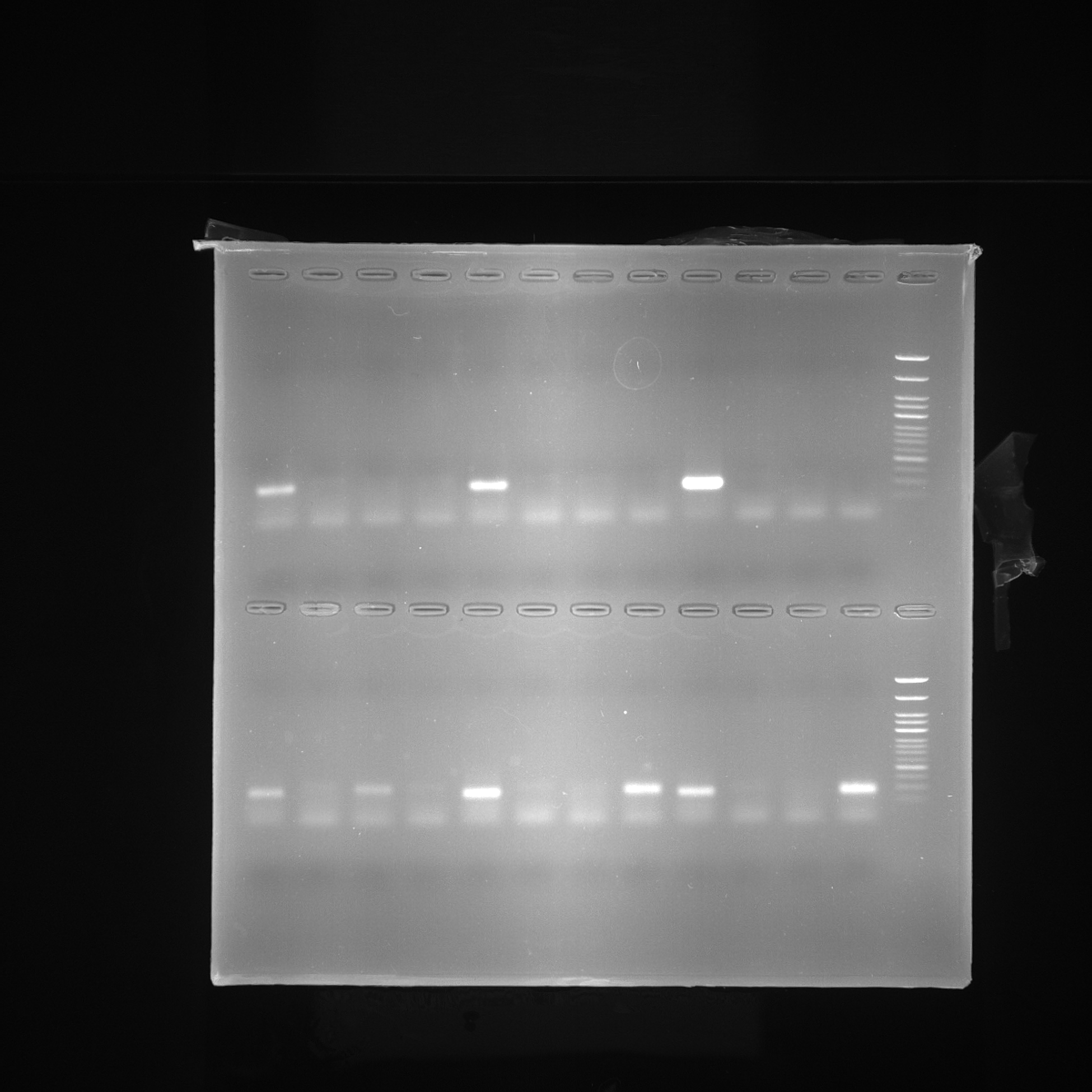

Supplement: Supplementary file 1 [file biomolecules-15-01507-s001.zip › Fig. 7.Tif]
